# Artificial intelligence (AI)-Enabled behavioral health application for college students: Pilot study protocol

**DOI:** 10.1371/journal.pone.0335847

**Published:** 2025-10-30

**Authors:** Edlin Garcia Colato, Aijia Yuan, Sagar Samtani, Bernice A. Pescosolido

**Affiliations:** 1 Department of Health and Wellness Design, Indiana University School of Public Health, Bloomington, Indiana, United States of America; 2 Department of Operations and Decision Technologies, Kelley School of Business, Indiana University, Bloomington, Indiana, United States of America; 3 Department of Sociology, College of Arts and Sciences, and the Irsay Institute, Indiana University, Bloomington, Indiana, United States of America; Firat University, TÜRKIYE

## Abstract

Given the prevalence of depression among young adults, particularly those aged 18–25, this study aims to address a critical need in higher education institutions for proactive, private, automated mental health self-awareness. This study protocol outlines how a mobile phone application will leverage sensor signal and survey data to develop an automated screening tool for depressive behaviors. By analyzing sensor-based behavioral data through deep learning techniques, the proposed study seeks to identify students exhibiting depressive symptoms and their specific behaviors. Approximately 1,000 first-year undergraduate students (age 18 and above) will be recruited from two public US universities, one in the Midwest and one in the Southwest. For the midwestern university, there will be 11 surveys (baseline, nine follow-ups, and an endline) collected throughout a single academic year (2024–2025). However, at the southwestern university, only nine surveys will be administered during a semester. Simultaneously, sensor-based behavioral data on behaviors such as physical activity, social interactions, and sleep will be continuously collected passively. The main analysis will focus on understanding the relationships between human behaviors captured by sensor-based behavioral data and self-reported mental health surveys. Machine learning and deep learning algorithms will be used to uncover key behavioral patterns most indicative of mental disorders such as depression.

## Introduction

Young adults, who comprise most college students in US universities, are the age group with the highest prevalence of depression. A 2022 report by the Substance Abuse and Mental Health Services Administration (SAMHSA) indicated that 18–25-year-olds had the highest reported percentage (18.6%) of major depressive episodes (MDE), of which nearly half did not receive treatment [1]. MDEs can interfere with their progress and even completion of higher education. Although affordability of higher education is a top reason for leaving college, 1/5 of students who withdrew from college also cite mental health as a top reason [2]. Consequently, higher education institutions bear a significant responsibility to proactively identify students who need to be referred to traditional on-campus and/or newer virtual (e.g., TimelyCare) services. To identify individuals at high risk, screening tools such as the Patient Health Questionnaire (PHQ) are typically provided to the students when arriving at their appointment.

Despite their prevalence, conventional assessments for screening possess several key limitations that necessitate carefully designed alternative approaches. First, such assessments require individuals to recognize key behavioral patterns. However, some individuals are unable to identify such patterns within themselves. Second, and relatedly, producing results for assessments requires self-reports, which are subjective and can be prone to misreporting. Finally, self-report-based approaches are often infrequent and, therefore, miss moment-to-moment behavioral fluctuations that can be associated with mental health concerns. Recent literature has provided compelling evidence that sensor signals produced from smartphones and other wearable devices offer a viable and promising data source to effectively overcome the aforementioned limitations. Specifically, sensor signal data are captured continuously from an individual and can more objectively reflect behavioral patterns missed by self-reports. Recent advances in artificial intelligence (AI) have created new opportunities to improve the detection of mental health conditions by processing the high velocity and voluminous sensor signal data in ways that are more objective, continuous, and unobtrusive than manual or conventional analysis [3,4].

A growing body of research has demonstrated that AI models can achieve strong predictive performance by analyzing data from social media, smartphones, and wearable devices [5–7]. These innovations are particularly promising for conditions like depression, which are widespread but often underdiagnosed, and where scalable early detection methods can have meaningful impact [8]. At the same time, these advances raise important ethical concerns, including issues related to user privacy, data transparency, informed consent, and algorithmic bias [9,10]. In response, interdisciplinary collaboration among technologists, clinicians, sociomedical researchers, and public health experts, along with increasing international collaboration, is recognized as essential to guide the responsible development and use of such AI-enabled mental health tools [11,12].

Among various emerging data sources, sensor data offers a particularly promising avenue for the passive and scalable capture of human behavioral patterns [13]. Sensor signals from smart devices (e.g., iPhones) often reflect physical and social behaviors that could be analyzed for a more automated and efficient approach to help screen for individuals exhibiting behaviors more indicative of depression than those captured through self-reports [14]. Since many college-aged students have adopted smartphones, analyzing the data from the sensors on these devices could be a viable approach to automatically screen individuals exhibiting depressive behaviors. The evidence shows there is a high level of acceptability for digital mental health interventions among young adults [15]. Given the scale of data generated from these devices, this exploratory study aims to develop Artificial Intelligence (AI)-enabled techniques based on deep learning to automatically screen individuals exhibiting depressive behaviors based on the sensor signal data generated from smartphones.

First-year incoming students are of specific interest because of the high attrition rates they present, especially within the first six weeks of their college journey as they adjust to the new physical and social environment of a residential campus. This period, often marked by significant transition and adjustment challenges, influences student retention [16]. By synergizing technology with mental health awareness, we aim to enhance student well-being, advance retention efforts, and contribute to broader mental health discourse.

### Study objectives

1 To examine the relationship between student behavioral patterns – captured through passive smartphone sensor signals – and their self-reported mental health status using survey data.
2 To develop and apply AI-enabled analytics to automatically:
  a) Identify individuals exhibiting symptoms consistent with depression.
  b) Detect specific behavioral markers (e.g., reduced mobility, irregular sleep patterns) associated with depressive symptoms.
  c) Assess how these behavioral patterns correlate with the student’s understanding of mental health information.
3 To design and evaluate machine learning (ML) and deep learning (DL) models for analyzing smartphone sensor data, with the goal of creating scalable, automated tools for early detection of depressive behaviors.

## Materials and methods

### Study design

This is a longitudinal pilot study that will assess the role of technology in helping students understand their current mental health status. The content found here is from a study protocol; therefore, no data are included. The approach to meeting these objectives is to collect data via a mobile application.

### Ethical approval

The Human Subjects Office of Indiana University’s Human Research Protection Program approved this study protocol and the related amendment (19663).

### Participants and setting

A non-probability sample of incoming first-year undergraduate students from two US public universities will be recruited, one in the Midwest (University A) and another located in the Southwest (University B). Eligibility criteria are twofold: participants 1) must be a first-year undergraduate student, and 2) must be at least 18 years old. If the prospective participant does not possess an iPhone they will be excluded from the study because the mobile phone application is currently only compatible with the iOS system. Per university information technology internal reports, approximately 95% of the student body at University A utilize an iPhone, which allows for expansive reach.

### Sample size calculation

Drawing from the relevant prior literature on attrition rates for longitudinal studies, which range between 30% and 70% [17], a 40% attrition rate for this study is anticipated. To account for this, approximately 1,000 participants will be recruited. This conservative estimation allows for offsetting the anticipated data loss, ensuring a robust dataset of at least 500 active participants by the end of the data collection period (December 2025).

During each study activity, participant engagement will be reviewed and documented for attrition patterns. Response patterns will be monitored and additional contacts will be made for respondents who do not respond to or answer follow-ups. Additional incentives will be offered for completion. This approach allows for an assessment to determine whether attrition is random or systematic. Sensitivity analyses will be conducted to evaluate the robustness of our findings under various assumptions about missing data. These analyses could include techniques such as multiple imputation or complete-case analysis to assess the potential impact of attrition on study outcomes. To minimize loss to follow-up, participants who choose to opt-in will receive reminders via email, text, or application notifications about when surveys open and close.

### Recruitment

At University A, the study will be advertised during new student orientation and welcome week campus activities. Recruitment will occur during tabling events where interested participants can approach us for additional information. The table will include a poster with eligibility criteria and a brief explanation of the study with a QR code to download the mobile application named Mental Health AI-Pal (MHAI-Pal), pronounced “my pal,” to their iPhone. The application will have the prospective participants confirm their eligibility criteria before proceeding to the consent form. However, at University B, recruitment will be conducted in a classroom setting at the beginning of the spring (January 2025) and fall (September 2025) semesters. The recruitment information will be broadcast via PowerPoint and our team will provide technical assistance on site.

Voluntary informed consent will be collected via an electronic modality using the mobile application. Consent forms will be provided digitally for prospective participants to read prior to enrolling. The research team will review the consent form with the prospective participant and answer any questions at the booth during enrollment. A digital copy of the consent form will be available for individuals who need more time to deliberate about their participation. Participation in the study is entirely voluntary, and every effort will be made to ensure that prospective participants understand the risks and benefits of participation and what will be asked of them if they decide to participate. Accordingly, they may take as much time as needed to peruse the informed consent documents before providing digital confirmation consent to participate in the study. Consent is documented in the mobile application. Participants who select ‘yes’ to consenting will then be able to create a profile and set up the data collection settings that will begin once classes start in August 2024 at University A. Recruitment began on 10/06/2024 at University A and will continue on an ongoing rolling basis until 07/09/2025. However, at University B, recruitment will be conducted in a classroom setting at the beginning of the spring (January 2025) and fall (September 2025) semesters.

### Data collection

At University A, the sensor-based behavioral data collection will start one week before the fall 2024 semester and continue until a week after the conclusion of the Spring 2025 term. Data collection at University A is anticipated to conclude by May 2025, and results will follow shortly after. This will capture the participants’ behavioral changes, accommodating the seasonal shifts that are known to influence mood changes. Notably, the study’s start and end weeks correspond to the participants’ moving-in and moving-out periods, providing additional behavioral contexts tied to these significant transitional phases of a first-year student’s life. On the other hand, at University B, the data collection will begin at the start of a semester and conclude at the end of that same semester (spring and fall 2025). Participants will be enrolled for only a single semester. Data collection at University B will conclude in December 2025, with results available during the second quarter of 2026.

**Fig 1:**
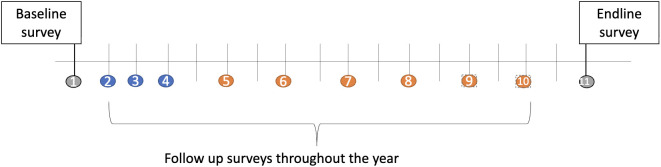
Data collection timeline at University A for proposed MHAI Study.

The study activities at University A include downloading the study application to the participant’s iPhone during new student orientation/welcome week recruitment events to initiate the passive mobile sensor data collection beginning the week before the start of the fall 2024 semester. The baseline survey (15 minutes) is distributed via the study’s mobile application and is completed during the first week of the fall semester (Fig 1). Following the baseline survey, participants complete bi-weekly behavioral surveys (9 total, approximately 2 minutes to complete) leading up until the endline survey (15-minutes) distributed one week following the end of the spring semester (May 2025).

**Fig 2:**
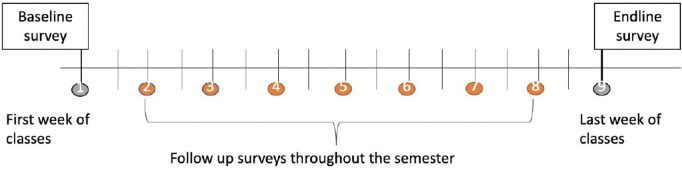
Data collection timeline at University B for proposed MHAI Study.

At University B, data collection will be for one semester, with a reduced amount of follow-up surveys, seven instead of nine, made available every two weeks, as illustrated below (Fig 2). Participants will be provided with the following surveys: the baseline during the first week of the semester, seven follow up surveys every two weeks, and finally the endline during the final week of the semester.

The data collection will encompass a variety of methodologies, each of which is detailed below.

### Survey instruments

*Baseline and Endline Surveys* This baseline survey will encompass the Flourishing Scale (FS-8), PHQ-9, GAD-7, Perceived Stress Scale (PSS-10), Loneliness Scale-3, Pittsburg Sleep Quality Index-Brief (B-PSQI), and the mental health-promoting knowledge (MHPK-10) instrument (Table 1). An identical endline survey is administered via the mobile application at the end of the data collection period.

**Table 1:** Measurement tools used in surveys.

Item measured	Measurement tool
Depression	The PHQ-9 is comprised of nine questions aimed at screening for depression. Overall scores range from 0–27. Severity of symptoms is associated with higher overall scores [18,19].
Anxiety	The GAD-7 questionnaire screens for severity of anxiety [18,20]. Total scores range from 0–21. The higher the score, the more severe symptoms of anxiety.
Positive mental health	FS-8 – The 8-item flourishing scale measures self-perceived success using a 1–7 scale per question. A single overall score is generated, with 8 being the lowest score possible and 56 the highest [21].
Stress	PSS-10 – The 10-item Perceived Stress Scale measures individual stress levels. Each question has response options 0–4. A total overall score ranges between 0 (no stress) to 40 (high perceived stress) [22].
Loneliness	Loneliness Scale-3 – The three-item loneliness scale is a tool used to measure to what extent an individual feels lonely. Scores range from 3–9; higher scores indicate an increased level of loneliness [23].
Sleep	B-PSQI – The brief version of the PSQI is a 6-item tool that measures sleep quality. Response options per question are 0–3. Total scores range from 0 to 18. Higher scores are associated with worse sleep [24].
Mental health literacy	MHPK-10 – Measures individual-level knowledge about obtaining and maintaining good mental health [25].
Social network	Social network battery – Captures respondent’s social network and relationships [26].

Recognizing the initial six weeks of the semester as a period with heightened attrition risk for first-year students, the data collection strategy is tailored to capture this significant phase. Specifically, during the first two months of the semester at University A, survey responses will be gathered bi-weekly (for three biweekly surveys) to capture potential fluctuations in mental health status as students transition into university life. Subsequently, starting in November 2024, the frequency will be reduced to a monthly basis (total of 6 monthly surveys), synchronizing with the anticipated stabilization of students’ circumstances. Push notifications will be sent via the mobile application to prompt students to complete follow-up surveys. Each notification grants students a 24-hour window for questionnaire completion, striking a balance between prompt data collection and accommodating diverse schedules. These follow-up surveys are estimated to take approximately two to five minutes. In addition to the PHQ-9 and GAD-7, the third follow-up survey will include a social network battery [26] to determine the types of relationships formed during the first semester of college. This will add approximately 10–15 minutes to the completion time for only this follow-up survey. Approximately two months later, the fifth follow-up survey will include questions about the first-year experience. This includes questions about the top three stressors on campus and their impact on their future at the university. This will add approximately two to five minutes to the estimated completion time for this follow-up. Since survey scores are not automatically screened and are reviewed months after participant submission and respondents are anonymous, links to campus and national resources (e.g., 988, Crisis Text Line, TimelyCare) are provided at the end of every survey and are always available to access on the mobile application. Participants are fully informed about the nature of the study, the use of their data, and the non-clinical role of the screening tools during the consent process.

### Sensing data collection

The collection of mobile sensor-based behavioral data will be facilitated through a pre-developed mobile application. This approach captures participants’ physical and social behaviors, presenting a more objective and efficient method for identifying individuals exhibiting depressive behaviors when contrasted with human assessments or self-reports. The sensor-based behavioral data intended for collection and the corresponding iOS frameworks is summarized for reference (Table 2). Data is collected at regular intervals while the phone is charging.

**Table 2:** Passive sensor-based behavioral data types and descriptions, MHAI Study.

Sensing Data	iOS Framework	Description
Accelerometer data	CoreMotion	Provides information about movement and velocity changes
Gyroscope data	CoreMotion	Provides information about rotation and orientation changes.
GPS location	CoreLocation	Provides the geographical location of the students.
Step count	HealthKit	Indicates the number of steps the student takes.
Distance traveled	HealthKit	Represents the total distance covered by the student.
Calories burned	HealthKit	Provides an estimated number of calories the student burns.
Sleep duration	HealthKit	Indicates the total length of sleep.
Sleep interruptions	HealthKit	Represent the number of interruptions during sleep.
Sleep intensity	HealthKit	Indicates the depth or intensity of sleep.
Light exposure	HealthKit	Represents the amount of light exposure.
Accelerometer data (during sleep)	CoreMotion	Provides physical activity data while sleeping.
Sound level	AVFoundation	Indicates the ambient sound levels in the user’s environment.
Nearby Bluetooth signals	CoreBluetooth	Represent signals from other Bluetooth devices in proximity.

### Compensation

To acknowledge participants’ contributions, each University A participant will receive a “swag bag” (see detail below) upon enrolling in the study. Compensation will be offered for completing the baseline, nine follow-up surveys, and one endline survey between August 2024 and May 2025. Participants who complete all study procedures will be eligible for a total compensation of $65 ($10 for baseline, up to $45 for the follow-up surveys, and $10 for the endline survey). Compensation will be dispersed via electronic gift cards in increments of $10 or $15). The maximum possible compensation for participants who complete all study procedures is $65, with decreasing compensation for incomplete study procedures. In addition to the financial incentive, five U Bring Change to Mind (UBC2M; an IU peer-to-peer mental health program) items will be distributed, as a *swag bag*, to participants upon initial sign-up, mobile application download, and onboarding procedure completion. The UBC2M initial enrollment incentive will include a backpack, back scratchers, shoelaces, sunglasses, and a t-shirt (items included depending on availability).

University B course instructors will provide extra credit to their students for engaging in this research. Instructors will also ensure there are alternative opportunities for extra credit for the students who a) either do not meet the eligibility criteria and are interested or b) meet the criteria but are not interested in enrolling in the study.

### Data processing

#### Statistical analysis.

As part of the comprehensive study design, a robust ground truth and a gold standard dataset is established following the methodologies used in most prior relevant studies. For this, PHQ-9, GAD-7, FS-8, and PSS-10 scores provide a baseline measure of students’ mental health, encompassing depression, anxiety, well-being, and stress, respectively. Integrating the survey scores into the gold standard dataset alongside the sensor-based behavioral data allows for nuanced insights into how various mental health characteristics manifest in real-world behaviors. Specifically, computed survey scores will serve as input features (e.g., baseline mental health status) to support descriptive analyses that examine relationships with demographic variables and help us better understand our participant pool. They will also serve as gold standard outcome labels (e.g., PHQ-9 for depression level prediction), used to train models on behavioral signals derived from sensor data. This comprehensive approach will enable bridging the gap between self-reported measures and objective behavioral patterns, enriching the depth and accuracy of the study’s findings.

Passive sensing data is pivotal as independent variables for monitoring sleep, steps, and other health behaviors. The focus includes a broad range of sensor signals, from physical activity data to social interaction indicators. The richness and granularity of these data provide a holistic view of the participants’ behavioral characteristics in their day-to-day lives, facilitating a unique moment-by-moment understanding. The key dependent variable in the model is whether a student is at a high risk of exhibiting behavioral or mood characteristics of depression. The primary aim is to build an efficient predictive model that uses the collected sensor data to generate early and accurate predictions of students’ mental health states. This is a step towards proactive mental health care, enabling early interventions for individuals who are at high risk.

To identify students at elevated risk for depression based on sensor data, a tiered analytical approach involving both classical machine learning (ML) models and deep learning (DL) models will be implemented. First, classical ML benchmark models, including logistic regression (LR), decision trees (DT), random forest (RF), K-nearest neighbors (KNN), and support vector machines (SVM) will be evaluated. These models will assess the predictive power of aggregated features derived from sensor data collected throughout the study period. In parallel, an exploration will be conducted regarding the DL models that are better suited for capturing non-linear relationships and temporal dependencies in multivariate time series data. Specifically, the following will be implemented: artificial neural networks (ANN), 1D convolutional neural networks (1D-CNN), recurrent neural networks (RNN), long short-term memory networks (LSTM), gated recurrent units (GRU), 2D convolutional neural networks (2D-CNN), and bidirectional LSTMs (Bi-LSTM).

Building upon these architectures, the Multiview-based Agreement Self-Attentive Model (MV-ASAM), a novel attention-based framework designed to detect depressive behaviors from multiple sensor modalities will be developed. MV-ASAM learns view-specific representations for each modality (e.g., activity, sleep, social interaction) and uses an agreement-based attention mechanism to identify features that are both predictive and consistent across views. This setup enables the model to weigh important behavioral signals within each modality while capturing cross-view patterns, improving both detection performance and interpretability.

All sensing data types summarized in Table 2 will be used as input features in these models. This includes physical activity (e.g., accelerometer data, step count, distance traveled), sleep patterns (e.g., sleep duration, interruptions, intensity), and social interaction indicators (e.g., Bluetooth proximity, sound levels). These features were selected because they reflect key behavioral domains, such as activity, sleep, and sociability, that align with core diagnostic criteria for depression as outlined in the DSM-5. Rather than limiting the feature space, we intentionally include a comprehensive set of sensing variables to better capture the multifaceted nature of depressive behaviors and to allow the models to learn meaningful patterns across different behavioral dimensions.

Missing sensor data will be addressed through short-window imputation strategies (e.g., forward or backward filling, median imputation across days), and participants with excessive missingness will be flagged for exclusion in sensitivity analyses. For missing survey responses, pairwise deletion or multiple imputation will be applied depending on the extent of the missing data. Confounding variables such as gender, race/ethnicity, and baseline mental health status will be included as covariates in the models when appropriate. In addition, class imbalance will be addressed using oversampling techniques such as SMOTE during model training.

Model performance will be evaluated using standard classification metrics, including accuracy, precision, recall, specificity, F1 score, and the area under the Receiver Operating Characteristic (ROC) curve. To assess generalizability and minimize overfitting, a 10-fold cross-validation strategy will be adopted. To further understand the intricate relationships between different behavioral characteristics and depression, regularly feature importance analysis will also be conducted throughout the year and each semester. This analysis will be done through the DL-based models (e.g., MV-ASAM), designed to identify the most important input features (i.e., independent variables) that contribute to the dependent variable (i.e., outcome).

### Data management plan and data security

Primary data will be collected using mobile phones (iOS) and stored electronically in relational databases (MySQL) for analysis. The data will be managed and processed using Python, a widely used sensor signal data analysis programming language. The storage location will be backed up automatically every two weeks. Quality assurance steps will include testing the database by the study team before moving to production mode. The following quality control methods will be used: random checks of accuracy, extraction, and cleaning of data that will be used for analysis, as well as other standard data pre-processing steps taken in machine learning literature. Quality assurance measures, such as built-in range checks and database testing by the study team, will be implemented to ensure the accuracy and reliability of the collected data. Additionally, regular data extraction and cleaning processes will be performed at regular intervals, typically every two months, to prepare the data for analysis.

To ensure participant privacy and protect the confidentiality of their data, stringent measures will be implemented:

- **Data Transmission:** Upon collection, data will be stored locally, adhering to Apple’s encryption standards before transmission. This encrypted data is transmitted using SSL certificates to the Jetstream server, where a two-step encryption process occurs.
- **Encryption:** All participant data will be encrypted during storage. To further protect participant privacy, personally identifiable information (PII) will be removed or anonymized via the encryption process and, when retrieved, will be information devoid of PII (e.g., usernames will be retrieved as generated study IDs with no PII). The protection of participant privacy by removing or anonymizing PII will be implemented at various stages of the data flow. Data will also be encrypted upon arrival at the Jetstream server and re-encrypted before being sent to the backend MySQL server for storage, which utilizes AES encryption.
- **Access Control:** Access to the data will be restricted to authorized study personnel. Only individuals with a legitimate need to access the data for research and analysis purposes will be granted permission. Permission to view data will be given via a key and only made available to the Principal Investigator (PI) and limited designated research members. This access control ensures that only the PI and lead graduate students will be able to retrieve the data, preventing the identification of individuals and maintaining their confidentiality throughout the study.

## Discussion

### Limitations

Limitations of the study design must be acknowledged. One is that the recruitment modality of in-person tabling activities at social events will inherently exclude or deter individuals who are uncomfortable or unable to approach the table and those who simply avoid attending campus events. Similarly, recruitment from a classroom limits diversity of student majors as most students in a specific course share an interest in the enrolled class to complete their degree. This limitation and the biases introduced from a convenience sample will be acknowledged in future data analysis. Strategies to address the biases in future studies will be developed post-analysis once it is determined which key demographic groups were significantly missing from the study. An example strategy includes targeted recruitment efforts in classrooms for courses offered to specific majors that were missed as well as partnering with student organizations that serve underrepresented groups. Another limitation of this study is that as a convenience sample focused on first-year college students with an iPhone, the findings from this study design may not be generalizable to the general public, older age groups, or people who own a phone that is not an iPhone. However, this is a pilot study from which improvements and resolutions to these limitations will be addressed in future iterations that include an expansion of the scope of eligible participants. A benefit of the iPhone selection for this study is that the majority of the population at the selected campuses use an iPhone rather than an Android device, which provides further reach in participants for this sample compared to having developed an Android-based application. Future studies at different college campuses need to take into consideration which type (iOS or Android) system is most prominently used, if only one system is available, or if resources allow, opting to create a mobile application that is compatible with both.

Another limitation is the level of prioritization participants will have for the study compared to their degree-dependent activities. Undergraduate participants are navigating their course schedules and other extracurricular activities, which will inevitably be prioritized over their engagement in the study. Therefore, anticipated challenges for this study include managing and successfully retaining enrolled study participants for the academic year-long survey. To assist the participants, the MHAI-Pal study team will send participants notifications of pending surveys via email, push notifications in the application, and short message service (SMS) text (if opted in). Pre-notifications in the form of emails and SMS have shown moderate to significant improvements in increased survey response rates [27,28]. Incentives and reminders together work towards increasing retention of participants [29]. The backend will monitor the progress of raw counts of completed tasks to help coordinate when additional reminders are needed.

### Dissemination plan

Following the conclusion of the pilot study (spring of 2026), results will be submitted as posters, conference abstracts, and peer-reviewed journals for consideration at public health, sociology, and information systems venues. Findings will also be shared with stakeholders, the research participants, and collaborators. Plans to disseminate the findings with research participants will consist of small group sessions to obtain their feedback on the findings. Dependent on participant input, findings may be shared with mental health services personnel at the universities. Keeping in mind this is a pilot study, and the feasibility of the study is being tested, therefore future study iterations might be more appropriate to share with broader university services such as counseling centers and student affair professionals.

### Plans for amendments

Amendments to the protocol are anticipated. In particular, the first amendment is focused on expanding the sample to include a more diverse student body from other universities. This amendment required an agreement with the Human Subjects Review Boards from University B and the instructors who can provide student access for the study team to conduct recruitment efforts. Further amendments may be considered to either increase the participant incentives (if available) or to improve communication efforts with the participants.

### Possible impact

This pilot study aims to leverage the sensor-based behavioral data from consenting individuals to advance personalized mental health awareness using AI tools. By creating an individualized tool tailored to the users’ unique behaviors, there could be a future in which each person can adjust their behavior based on their data rather than suggestions from the averages of aggregated data from other people. Currently, the majority of mental health tools aimed at the individual are focused on self-care, which have acute results. Individual behavior changes that have contributed to the symptoms are not being addressed. Therefore, this could have the potential to increase individual mental health literacy while receiving automated suggestions based on their actions.

At the same time, the implementation of such personalized systems still faces several challenges. Continued user engagement and integration with existing support structures remain key concerns in real-world deployment. Existing work in passive sensing has shown the feasibility of detecting depression using smartphone and wearable data [30,31]. However, broader generalization requires further validation across more diverse populations and longer observation periods. Additionally, identifying individuals at risk is only the first step; effective intervention strategies following detection must be carefully developed in collaboration with clinical experts to ensure these tools provide meaningful support in addition to continuous monitoring. Building on these foundations, this study aims to explore how interpretable AI-enabled tools can support proactive mental health monitoring in a scalable and responsible manner.
